# Associations between hospital characteristics and patient satisfaction in Germany

**DOI:** 10.1111/hex.12485

**Published:** 2016-07-22

**Authors:** Rike Antje Kraska, Marcel Weigand, Max Geraedts

**Affiliations:** ^1^ Institute for Health Systems Research School of Medicine Faculty of Health Witten/Herdecke University Witten Germany; ^2^ Weisse Liste gGmbH Berlin Germany

**Keywords:** health services research, hospital performance, patient satisfaction, report cards

## Abstract

**Background:**

The patient perspective is increasingly recognized as a central pillar of quality in hospital care. International evidence suggests that an array of interacting factors may influence patient satisfaction with hospital care, whereas only a few studies have examined the impact of hospital characteristics.

**Objective:**

To explore which hospital characteristics exert an influence on patient satisfaction with inpatient care.

**Design:**

Cross‐sectional study using secondary data.

**Setting and participants:**

A total of 999 hospitals in Germany with 300 200 patient surveys from 2013 formed the study population. Patient satisfaction was surveyed using the Patients’ Experience Questionnaire, and hospital characteristics were extracted from mandatory quality reports. Only hospitals with at least 75 surveys were included in the analysis.

**Main variables studied and main outcome measures:**

Four dimensions of patient satisfaction (medical care, nursing care, organization and overall impression) were studied as the outcome measures. Region, profit orientation, size, staffing per bed and quality scores were considered possible influencing hospital characteristics. We performed risk‐adjusted multivariate analyses.

**Results:**

All of the characteristics had a significant influence on the patient satisfaction dimensions (*P*<.05), and patients in East Germany, in small hospitals or in not‐for‐profit hospitals, were more satisfied. Additionally, more staffing per bed as well as a better process and outcome quality were associated with more satisfied patients.

**Conclusion:**

Structural and quality characteristics of hospitals have a significant impact on patient satisfaction. This association confirms that patients are sensitive to important hospital quality measures and reinforces the consideration of patient satisfaction as an indicator of the quality of care.

## 1 Introduction

Quality assurance and improvement of hospital care are crucial factors for an efficient health‐care system. Numerous countries underline this central importance by means of laws, reforms and initiatives. In Germany, for example the Hospital Structures Act, which came into force in January 2016, includes hospital quality or care as a criterion for hospital planning and hospital financing.^1^ As dimensions of the quality of hospital care, clinical safety and effectiveness are most frequently mentioned, although patient satisfaction is increasingly perceived as an additional quality dimension.^2^, ^3^

Patient satisfaction can provide valuable and unique insights into daily hospital care and is widely accepted as an independent dimension of quality of care because an evaluation of patient satisfaction includes “internal” (inward‐looking) aspects of hospital care, which often remain unrecorded, such as communication, empathy or interaction.^2^, ^4^, ^5^, ^6^ Numerous studies and systematic reviews demonstrate, however, a positive association between the subjective patient perspective and clinical safety and effectiveness, and they demonstrate that patient satisfaction reflects various dimensions of the quality of care.^2^, ^4^, ^7^, ^8^, ^9^, ^10^, ^11^

Thus, it comes as no surprise that the measurement of patient satisfaction is frequently used as a tool to improve quality of care.^8^, ^12^ International studies also suggest that the continuous evaluation and publication of patient surveys may complement public reporting on clinical outcomes and process quality in assisting patients in choosing a hospital and serving to improve the quality of hospital care on a long‐term basis.^7^, ^8^ Some countries have already introduced nationwide surveys and have published subjective patient perspectives of inpatient care, whereas in many cases, hospitals collect data on patient satisfaction themselves and use this information for internal quality management. However, some studies note that, so far, few hospitals actually use patient surveys as a basis for measures to improve quality, and very little information is available about feedback systems to improve quality of care based on patient surveys.^7^, ^13^ One possible reason for this deficit is that factors influencing patient satisfaction have been insufficiently researched up to now.^2^, ^7^, ^13^

Many studies explore the influence of demographic factors and patient attributes on patient satisfaction and often demonstrate divergent findings.^6^, ^13^, ^14^, ^15^ It is commonly accepted that patient perceptions are influenced by sociocultural and socio‐economic factors and that a low level of education or a better state of health, for example, may result in a more positive rating for satisfaction.^6^, ^14^, ^15^ However, such frequently explored patient attributes are unsuitable as reference points to improve quality of hospital care and patient satisfaction.^13^ Instead, they indicate the relevance of using a risk adjustment that should be performed to compare patient satisfaction ratings between hospitals and is based on patient attributes. Few studies have explored the influence of hospital characteristics on patient satisfaction.

Nevertheless, there is a broad consensus that patient satisfaction is strongly influenced by the quality of nursing.^6^, ^13^, ^16^, ^17^ In a German study on the determinants of patient satisfaction in inpatient care from 2011, Schoenfelder et al.^6^ concluded that satisfaction with nursing has the most significant impact on overall satisfaction. Additionally, in a study on patient safety, satisfaction and hospital quality in 12 European countries and the United States, Aiken et al.^16^ found that – considerable differences in survey results between countries notwithstanding – nursing, in general and across countries, exerts a significant and strong influence on patient satisfaction. The authors also demonstrated that better staffing ratios (the number of staff per patient) and a better work environment were associated with a higher quality of care and higher levels of patient satisfaction.^15^

Moreover, several studies found that clinical quality characteristics also influence patient satisfaction.^2^, ^7^, ^8^, ^10^, ^11^, ^17^ In a systematic review on the links between the patient experience and clinical safety and effectiveness conducted in 2012, Doyle et al.^2^ concluded that the subjective patient perspective is consistently positively associated with clinical effectiveness across a broad range of diseases, study designs, study groups, environments and outcome measures. The authors underlined, however, a great and continuing need for research into the association between patient satisfaction and clinical performance.^2^

At the international level, and in Germany in particular, very little and insufficient information is available on the links between hospital characteristics and patient satisfaction with inpatient care. However, these findings are indispensable for improving patient satisfaction and the accompanying improvement in the quality of care. The aim of the study was to determine which structural and qualitative hospital characteristics exert an influence on patient satisfaction in inpatient care in Germany. Building on this, the authors expect that the findings from the study and the identification of determinants of patient satisfaction will help hospitals to further develop and improve their quality of care.

## 2 Methods

The study was designed as a cross‐sectional study based on secondary data from the year 2013. We used patient surveys from the “Weisse Liste/White List” for patient satisfaction, as well as mandatory hospital quality reports that cover structural and quality characteristics.

### 2.1 Survey of patient satisfaction

Since 2011, in Germany, the project “Weisse Liste/White List” has performed surveys on patient satisfaction with inpatient stays with the primary objective of assisting patients in selecting hospitals through the online publication of comparative survey results. The patient satisfaction data are jointly surveyed by two large statutory health insurers (AOK and BARMER GEK, covering a total of approximately 33 million insures, and 47% of all statutorily insured) and are again prepared and published by the “Weisse Liste/White List.” Raw data provided by the “Weisse Liste/White List” from surveys conducted in 2013 were exclusively considered and used for the purposes of this study.

The survey on patient satisfaction is designed as a post‐hospitalization survey in which patients are asked to retrospectively assess their experience with their hospital stay. In the course of the investigation period (2013), five anonymous survey rounds independent of the disease were conducted among patients previously discharged between 2 and 8 weeks. Only patients between 18 and 80 years of age at the time of discharge who had received inpatient care for at least 2 days were included in the study population (SP). If there were several hospitalization periods, the survey referred to the last hospitalization, and only one survey per year was permitted. Patients were excluded from the study population if they had specific reasons for discharge or transfer (e.g. transfer to a nursing home), nursing care needs, certain diagnoses (survey was an unreasonable burden on the patient, e.g. psychiatric main diagnosis), an employment contract with the insurer conducting the survey or residence abroad. Also excluded were all cases designated as childbirth. A total of 1 028 898 patients from 2013 were contacted by post, with a response rate of 39.7%.

The tool used for the survey was the Patients’ Experience Questionnaire (PEQ), a short standardized questionnaire comprising 15 questions, developed by the Bertelsmann foundation and Verein Outcome in Switzerland, and tested according to scientific methods.^18^ The questionnaire and explanations on the development and scientific evaluation of the questionnaire are available on the homepage of “Weisse Liste/White List.”^18^, ^19^ The PEQ covers four quality dimensions that were also addressed in this study: (i) satisfaction with medical care (questions 1–4), (ii) satisfaction with nursing care (questions 5–8), (iii) satisfaction with the organization and service (questions 9–13) and (iv) recommendation/general satisfaction (question 14). Participants in the survey were also asked to note their age and gender. All questions involved a standardized rating scale from 1 to 6, where “1” was the best possible rating and “6” was the worst. To determine patient assessments of each quality dimension, responses to individual questions were converted into a corresponding percentage value (1=100%, 2=80%, 3=60%, 4=40%, 5=20%, 6=0%), and the mean value was calculated using the questions referring to the respective quality dimension.

Merging of surveys with the respective hospital was performed via the hospital institution code (IC), which is used for reimbursement purposes. It must be noted that the IC cannot be considered to be a unique key variable.^20^ One hospital may have several accounting units, or several hospitals in a hospital group may use the same IC.^20^ A total of 408 460 patient questionnaires were returned for the investigation period, of which 94 671 surveys could not be merged with the hospital data due to the above‐mentioned reasons.

Of the 313 789 returned questionnaires that were uniquely merged to the hospital data, patients’ percentage ratings for the four quality dimensions were aggregated at the hospital level (1476 hospitals). Patient age, gender and specialist departments were used as potential risk factors for adjustment. Risk adjustment was performed via multivariate regression, where the factor for “observed satisfaction” in relation to “expected satisfaction” (taking adjustment factors into account) was calculated for all four quality dimensions and for each hospital. Accordingly, in direct comparison, a hospital with a factor of more than one may be considered to be a hospital with a better rating. Moreover, only hospitals with a minimum of 75 returned PEQs were included in the analysis. As a result, the analysed study population comprised 999 hospitals with a total of 300 200 patient surveys.

### 2.2 Hospital characteristics

Structured hospital quality reports in XML format from the reporting year 2013 provided by the Federal Joint Committee served as the data source for the structural and qualitative hospital characteristics. Quality reporting is mandatory since 2005 and comprises data on the structures and performance of the entire hospital or specialist departments as well as data on quality assurance in the form of quality indicators from the so‐called external quality assurance system. The following variables were extracted from the quality reports, processed and used as structural characteristics with a possible impact on patient satisfaction: (i) region, with the categories of north‐west, south, and east; (ii) hospital ownership, with the categories of public (non‐profit), charitable (non‐profit) and private (for‐profit); (iii) number of beds/hospital size; (iv) medical staffing per bed; (v) nursing staffing per bed; and (vi) therapy specialists per bed.

Three quality scores of hospital quality characteristics were calculated from the 295 quality indicators from 30 different clinical areas listed in the QRs in 2013. A detailed description of all of the quality indicators is available on the homepage of the AQUA Institute, which was responsible for administering the external quality assurance system (eQAS) in 2013.^21^ The calculated and analysed scores cover three quality dimensions: outcome quality (216 indicators), process quality (62 indicators) and indication quality, which assess the appropriateness of performing a procedure (17 indicators). It must be noted that not all of the hospitals necessarily had data for all of the quality indicators available. Because hospitals usually do not perform all of the procedures covered by the eQAS, different numbers of quality indicators were determined for each hospital. For the calculation of the quality scores, the upper 75% quantile was determined for each quality indicator across all hospitals and was established as the reference range. Subsequently, the ratio of quality indicators within the reference range was calculated for each hospital in relation to all of the individually indicated quality indicators in the respective dimension and was used as a quality score. Accordingly, the score was expressed as a percentage, of which a higher value indicates higher quality.

### 2.3 Analytical procedure

The structural and qualitative hospital characteristics were merged with the risk‐adjusted patient surveys at the hospital level via the IC, wherein unique assignability was possible.

In a first step, the aggregated dimensions of patient satisfaction were analysed descriptively, followed by a verification of the representativeness or a possible systematic bias in the study population. For this purpose, the structural characteristics, such as region, hospital ownership and size of the study population, were compared to the “basic data of hospitals” from 2013 provided by the Federal Statistical Office and examined for significant differences via chi‐squared tests.^22^ Hospital quality reports, on the other hand, were used for the characteristics of staffing and quality, and *t*‐tests were performed to compare the hospitals in the study population to those excluded from the sample. Dimensions of patient satisfaction were explored in the same manner, using all of the available patient questionnaires from 2013 as the source.

Correlations between individual structural and qualitative characteristics and patient satisfaction were examined in the next step with the help of analyses of variance, *t*‐tests and correlation analyses. Significant predictors were then entered into a respective multivariate linear regression model as independent variables, and the model was determined via the stepwise forward integration of statistically significant variables (*P*<.05). Categorical variables were integrated in the models with the help of dummy variables. The findings served to explore the adjusted strength of the correlation between individual factors and patient satisfaction. All analyses and evaluations were performed with the statistics program SPSS 22 (IBM, Armonk, New York, USA).

## 3 Results

### 3.1 Observed patient satisfaction

In general, all hospitals achieved a high level of patient satisfaction with inpatient care. Table 1 shows that the mean rating of patient satisfaction was approximately 81.5% in the four quality dimensions under consideration, of which “organization and service” achieved the lowest average rating of 79%. However, a standard deviation of up to ±6.5% and a range of up to 41% revealed clear differences in patient satisfaction between hospitals.

**Table 1:** Descriptive statistics of four dimensions of patient satisfaction with and without risk adjustment

Satisfaction with…	Mean		Standard deviation		Minimum		Maximum	
	%	O/E	%	O/E	%	O/E	%	O/E
…medical care	82.85	1.002	3.96	0.042	66.88	0.81	94.53	1.13
…nursing care	81.76	1.003	3.99	0.047	68.76	0.84	93.15	1.13
…organization and service	79.34	1.008	5.14	0.062	64.21	0.82	94.69	1.19
…/in general	82.43	1.004	6.52	0.075	57.12	0.69	98.1	1.21

%, mean percentage without risk adjustment; O/E, observed/expected with risk adjustment.

### 3.2 Representativeness of the study hospitals

A comparison of the study hospitals (SP) with all German hospitals (GP) revealed some deviations that might endanger the representativeness. As Table 2 shows, the proportion of small hospitals with <100 beds in all German hospitals was approximately 35%, but in the study hospitals, the proportion of small hospitals was only 10%. Because small hospitals often have private ownership, a different distribution of hospital ownership was observable in the study hospitals in comparison with all hospitals, and the number of private (for‐profit) hospitals was significantly smaller (24% SP vs 34% GP).

**Table 2:** Results of examination for representativeness of sample via chi‐squared test and *t*‐test

	Number		Sig. Chi‐squared Test
	Study hospitals	All hospitals in Germany^a^	
Hospital size			
≤100 beds	100 (10%)	693 (34.7%)	.000
>100 to ≤500 beds	722 (72.3%)	1036 (51.9%)	
>500 beds	177 (17.7%)	267 (13.4%)	
Hospital ownership			
Public (non‐profit)	346 (34.6%)	596 (29.9%)	.001
Charitable (non‐profit)	416 (41.6%)	706 (35.4%)	
Private (for‐profit)	237 (23.7%)	694 (34.8%)	
Region			
North‐west	397 (39.7%)	728 (36.5%)	.021
South	414 (41.4%)	922 (46.2%)	
East	188 (18.8%)	346 (17.3%)	
N	999	1996	

	Mean		Sig. *T*‐test
	Study hospitals	Excluded hospitals^b^	
Staffing per bed			
Medical staff per bed	0.46	0.35	.000
Nursing staff per bed	0.73	0.66	.000
Therapy specialists per bed	0.2	0.31	.000
Quality			
Outcome quality	61.6	63.4	.135
Indication quality	44.1	44.0	.966
Process quality	57.4	55.9	.185
Risk adj. patient satisfaction			
Medical care	1.00	1.03	.000
Nursing care	1.00	1.03	.000
Organization and Service	1.01	1.05	.000
General	1.00	1.04	.000

a All hospitals according to Federal Statistical Office.

b Recordable data for excluded hospitals based on structured quality reporting or data on patient satisfaction not included in the study population.

Differences between the GP and the SP were also found for staffing. As Table 2 shows, the study hospitals contained significantly more physicians per bed (mean 0.46 SP vs mean 0.35 GP) and significantly more nursing professionals per bed (mean 0.73 SP vs mean 0.66 GP). No significant differences between study hospitals and all German hospitals were found in terms of quality. A comparison of risk‐adjusted patient satisfaction indicated, however, significantly better ratings for those hospitals that had been excluded.

### 3.3 Results of the multivariate regression models

As Table 3 shows, the multivariate regression models revealed significant associations between all of the examined structural and qualitative characteristics and the patient satisfaction for all dimensions. The quality of the models in the four risk‐adjusted multiple linear regression models was classified as good with an explained variation of between 29% and 40%. The investigated structural and qualitative characteristics exerted an almost equal influence on the four dimensions of patient satisfaction, such that only small differences were found between the dimensions.

**Table 3:** Results of risk‐adjusted multivariate linear regression models for the four dimensions of patient satisfaction (significant results only)

	Satisfaction with medical care (*R*²=.289)		Satisfaction with nursing care (*R*²=.314)		Satisfaction with organization and service (*R*²=.4)		General satisfaction/recommendation (*R*²=.293)	
	β	Sig.	β	Sig.	Β	Sig.	β	Sig.
Hospital size (beds)	−.309	.000	−.315	.000	−.42	.000	−.348	.000
Region (Dummy variables vs East)								
North‐west	−.508	.000	−.507	.000	−.542	.000	−.472	.000
South	−.178	.000	−.262	.000	−.248	.000	−.188	.000
Hospital ownership [Dummy variables vs charitable (non‐profit)]								
Public (non‐profit)	−.101	.002					−.113	.001
Private (for‐profit)	−.118	.000	−.197	.000	−.152	.000	−.221	.000
Staffing per bed								
Medical staff per bed	.254	.000	.088	.026			.142	.000
Nursing staff per bed			.103	.005	.163	.000	.109	.003
Therapy specialists per bed	.138	.000	.176	.000	.145	.000	.171	.000
Quality								
Outcome quality			.135	.000	.107	.001		
Indication quality			−.071	.021			−.063	.033
Process quality	.106	.001	.071	.022	.06	.039	.116	.000

#### 3.3.1 Region

Apart from hospital size, region had the largest impact on patient satisfaction. Table 3 shows that in comparison with the south or east, patients in the north‐west of Germany were less satisfied with medical care (β=−.508; *P*<.000), with nursing care (β=−.507; *P*<.000), with organization and service (β=−.542; *P*<.000) and in general (β=−.472; *P*<.000). Hospitals in the eastern parts of Germany achieved comparatively higher ratings for patient satisfaction in all four dimensions.

#### 3.3.2 Hospital size

As Table 3 shows, hospital size also had a considerable influence on all dimensions of patient satisfaction; a growing number of beds was associated with a decline in patient satisfaction. Compared to small hospitals, larger hospitals received lower ratings for patient satisfaction specifically in the field of organization and service (β=−.42; *P*<.000).

#### 3.3.3 Hospital ownership

Private for‐profit hospitals generally received lower ratings for patient satisfaction in all dimensions compared to non‐private, not‐for‐profit hospitals. Specifically, in terms of general satisfaction (β=−.221; *P*<.000) and nursing care (β=−.197; *P*<.002), patients in private hospitals appeared to be less satisfied than those in not profit‐oriented hospitals. Hospitals with public ownership (not‐for‐profit), however, received lower ratings for satisfaction with medical care (β=−.101; *P*<.002) and general satisfaction (β=−.113; *P*<.001) compared to charitable hospitals (not‐for‐profit) (Table 3).

#### 3.3.4 Staffing

Higher staffing per bed was associated with higher levels of patient satisfaction. As Table 3 shows, a higher number of medical staff per bed was specifically associated with more patient satisfaction with medical care (β=.295; *P*<.000), as well as with higher satisfaction in general (β=.142; *P*<.000). It also appeared to have a weak correlation to satisfaction with nursing care (β=.088; *P*<.026). Higher numbers of nursing staff per bed were associated with higher satisfaction with nursing care (β=.103; *P*<.005), organization and service (β=.163; *P*<.00) and higher general satisfaction (β=.109; *P*<.003). A higher ratio of therapy specialists was associated with a higher degree of patient satisfaction in all four dimensions of patient satisfaction.

#### 3.3.5 Quality

Scores for process and outcome quality were positively associated with patient satisfaction. Process quality had a significant impact on all dimensions of patient satisfaction. For general satisfaction (β=.116; *P*<.000) and satisfaction with medical care (β=.106; *P*<.001) in particular, better process quality was associated with more satisfaction. On the other hand, better outcome quality was associated with more satisfaction with nursing care (β=.135; *P*<.000) and with organization and service (β=.107; *P*<.001). A minor correlation was found between the indication quality and patient satisfaction; here, better quality was associated with a lower level of satisfaction with nursing care (β=−.071; *P*<.021) and generally lower satisfaction (β=−.063; *P*<.033) (Table 3).

## 4 Discussion

Analyses of the associations between hospital characteristics and patient satisfaction in inpatient care indicate that structural parameters, such as staffing, as well as the characteristics of the process and outcome quality of hospital care are positively correlated with patient satisfaction. Although patients are already highly satisfied with inpatient care in German hospitals, the study revealed that better staffing ratios per bed and also better process and outcome quality are associated with increased patient satisfaction.

This positive association in terms of process and outcome quality applies to all of the analysed dimensions of patient satisfaction (satisfaction with medical care, nursing care, organization and service, and in general/recommendation). Similar results were reported by international studies, which indicated a positive association between clinical quality characteristics and patient satisfaction.^2^, ^7^, ^8^, ^10^, ^17^ The results of this study did therefore not confirm the opinion often expressed by critics that patient satisfaction may only be influenced by external factors such as hotel services.^4^ In general, the identified associations emphasize that the patient perspective must be considered to be an important quality criterion in hospital care.

Our exploration of the influence of staffing on patient satisfaction illustrates that a higher staffing ratio per bed is associated with more patient satisfaction. An analysis of patient safety, satisfaction and quality of hospital care by Aiken et al.,^16^ for example, also found that higher nursing staffing ratios per patient were associated with a higher level of patient satisfaction. However, our study confirmed only, in part, the general consensus that nursing care and specifically the nursing staff exert a strong influence on patient satisfaction.^16^ Our findings indicate, for example, that at least in Germany, the medical staff has a stronger impact on general satisfaction than the nursing staff. Compared to other countries, Germany has a more physician‐centred health system, where only a small proportion of nursing staff has an academic background. This could result in a higher confidence and a greater focus on the medical staff, which in turn leads to a higher impact of medical staff on patient satisfaction.

Another result of our research is that patients in larger hospitals report less satisfaction compared to those in small hospitals. This finding is supported by the significantly higher patient satisfaction in hospitals excluded from the study, which received less than 75 patient satisfaction surveys and therefore tend to be smaller hospitals. However, Tevis et al.^23^ explored the relationship between patient satisfaction and favourable surgical outcomes and noted that patients in larger hospitals were more satisfied. However, it is indisputable that larger hospitals offer a broader service portfolio and have a different patient distribution across diseases compared to smaller hospitals. The type of disease and the state of health can both significantly influence satisfaction ratings, so these differences in the patient distribution may have played an important role in the association between hospital size and patient satisfaction. Whether our risk adjustment for specialist department, patient age and gender was sufficient to counter the described effect cannot be determined with certainty and indicates a need for further research.

Although private for‐profit hospitals frequently are among smaller hospitals, our analyses revealed that patients in private hospitals are less satisfied with all dimensions compared to patients in not profit‐oriented hospitals. Additionally, Jha et al.^17^ demonstrated that profit orientation may have a negative impact on patient satisfaction in a study on patient perceptions of hospital care in the United States. Our study could not identify possible reasons for this finding. It can only be assumed that profit orientation is accompanied by compromised quality, with possible effects on patient satisfaction. Further analyses would therefore be required with a more specific focus on the relevance of hospital ownership and profit orientation.

Among all of the investigated factors that had a possible impact on patient satisfaction, the relation between regions and patient satisfaction had the strongest association, and hospitals in the east of Germany achieved better ratings. However, the available data do not permit a conclusion about whether patients in the east were actually more satisfied or gave better ratings in general due to regional cultural differences. Based on the experience of older patients with the health‐care system of the former German Democratic Republic, patients in the east of Germany might have a different point of view to evaluate their experience. The adaptation and associated improvement of comfort and medical care after the German reunification in 1990 could have lead to a general higher patient satisfaction in eastern Germany. Similar findings have been observed in international studies, which revealed regional differences in results from patient satisfaction surveys.^16^, ^17^ It is evident from these findings that region needs to be included as an adjustment factor in the comparison of patient satisfaction between hospitals and that there is a need for further research on reasons for regional differences in patient satisfaction.

Based on the inclusion and exclusion criteria and the resulting study population, our analysis can only present trends. Moreover, the distribution of hospital size and ownership in our study diverges from the actual hospital population in Germany: the study population comprises a smaller percentage of private for‐profit and small hospitals compared to the national average. However, our analyses reflect the results for hospitals with at least 100 beds, due to the high number of hospitals in the sample; but, our findings are not easily transferable to smaller hospitals. In view of the potential influence of patient attributes on patient satisfaction, a study of smaller hospitals that treat a comparably smaller number of patients and therefore exhibit a high variance may be difficult. Even in our study, it is impossible to state with certainty whether the conducted risk adjustment and the inclusion criterion of at least 75 returned patient questionnaires per hospital were sufficient to balance the possible effects of patient attributes, such as socio‐economic and health status. Further studies with a more specific risk adjustment in terms of patient attributes are indicated and would facilitate the inclusion of smaller hospitals.

On the basis of the available data concerning patient satisfaction and hospital characteristics, additional methodological limitations exist. The survey on patient satisfaction was performed by post in which patients were asked to retrospectively assess their experience. The difference in time intervals between treatment and completing the survey, that is between 2 and 8 weeks, may have resulted in bias. Two international studies about predictors of patient satisfaction suggested a possible significant but contrary influence of the interval between treatment and survey completion on patient satisfaction. While Jackson et al. noted that patient satisfaction was significantly higher for longer intervals between treatment and survey completion, Quintana et al. demonstrated exactly the opposite.^14^, ^24^ An improvement of the health status as well as a more critical point of view in a familiar environment might interact with patient satisfaction over time. Also, the general validity of data concerning hospital characteristics from quality reports provided by the hospitals themselves might be compromised, out of concern for public image and the competition among hospitals.^20^ It is difficult to state with certainty whether the observed quality indicator values reflect actual quality. The validity of data from hospital administration and accounting and self‐provided information must therefore be scrutinized in general. Moreover, the quality scores calculated and used in our study reflect a general impression of the quality of hospital care. The impact of quality indicators on patient satisfaction could be considered somewhat more specifically, in particular, at the level of specialist departments that we were not able to analyse on the basis of our secondary data. However, the association we detected suggests that a specific exploration might reveal an even stronger correlation.

A general problem with all patient satisfaction measurements is that the patient satisfaction rating is overall high. It is well‐known that the general trend exists to evaluate satisfaction too positively, whereby for patient satisfaction measurements, further promoting factors exist such as the relief about convalescence or the authority of the physician. These trends and factors might result in bias, and because of the overall high patient satisfaction rating, it might be difficult to examine differences between hospitals. Also, our study shows a high level of patient satisfaction with inpatient care in all quality dimensions. And although significant associations between hospital characteristics and patient satisfaction in our study were found, the differences of the patient satisfaction ratings between hospitals in absolute numbers are rather low.

Another general limitation is that there is no internationally standardized definition and usage of the term “patient satisfaction.”^4^, ^5^, ^13^ Although the term “patient satisfaction” has been repeatedly described as the differences between expectation and experience, it is often used synonymously with the term “patient experience.”^5^, ^13^ Additionally, the questionnaire used in our study (PEQ) contains general questions on patient satisfaction as well as on patient experience, which prevents a clear distinction between the terms. This difficulty reduces the comparability of studies and indicates a need for internationally harmonized definitions and usage of terminology.

In summary, the findings of our study suggest a correlation between structural and qualitative hospital characteristics and patient satisfaction in inpatient care, of which higher staffing ratios per bed and higher quality are associated with higher patient satisfaction. The positive association of the process and outcome quality with patient satisfaction emphasizes that the subjective patient perspective must be perceived as a significant quality criterion for inpatient care and can be regarded as an indicator of the quality of care in general. It would be advisable to include data on patient satisfaction in obligatory quality reporting to improve the quality of hospital care at the system level.

## Conflict of Interests

No conflict of interests have been declared.

## Source of Funding

This work received no specific funding.
